# A novel recovery algorithm of time encoded signals

**DOI:** 10.1186/1471-2202-14-S1-P130

**Published:** 2013-07-08

**Authors:** Dorian Florescu, Daniel Coca

**Affiliations:** 1 Department of Automatic Control and Systems Engineering, University of Sheffield, Sheffield, South Yorkshire, S1 3JD, UK

## 

**Figure 1:**
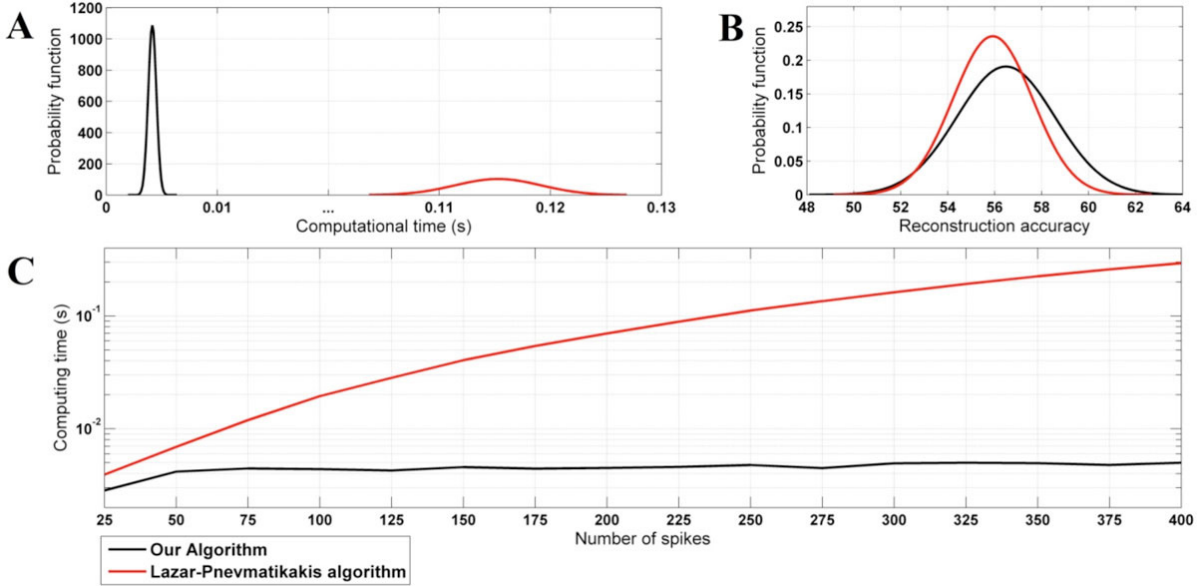
**Reconstruction performance comparison: **A **- computing time, **B **- accuracy (SNR), **C **- computation time as function of the number of spikes used in reconstruction**.

The integrate-and-fire (IAF) neuron model is a Time Encoding Machine (TEM), which maps analog signals into a sequence of strictly increasing time events. There are a number of reconstruction algorithms that ensure perfect recovery of bandlimited input signals from spike trains [1]. For signals that are not bandlimited, or when their bandwidth is unknown, the algorithms available [2] ensure that the reconstructed signal satisfies a consistency constraint, i.e., the reconstructed stimulus generates the same spike train as the original stimulus. As noted in [2], consistent reconstruction is more relevant and useful for recovering real sensory stimuli because a good estimate of the bandwidth is often not available. The existing algorithms that reconstruct the inputs encoded with TEMs exploit, in some form, the nonuniform sampling theory. Here we propose a novel consistent algorithm for signal reconstruction from spike trains, which involves solving an associated interpolation problem based on uniformly sampled data, and present theoretical results that underpin the proposed reconstruction method. While providing similar accuracy, our algorithm is faster and thus better suited for real-time processing, because our approach does not require recalculating the basis functions used in reconstruction for different sets of spikes. In addition, the new approach provides an alternative framework to study spike processing. To show the performance of our algorithm, we compared it to the best consistent reconstruction method available [2]. The input used was a periodic signal with randomly generated Fourier coefficients. The computation time and reconstruction accuracy of each algorithm were evaluated for 100 randomly generated input sequences. The estimated probability density functions corresponding to each performance index are shown in Figures 1A&B. The average computation time, plotted as a function of the number of spikes (Figure 1B), demonstrates that computing time is almost independent on the number of spikes. The simulations were carried out in Matlab on a 3.10 GHz Intel Single Core PC workstation.
